# Metabolic Reprogramming of Chemoresistant Cancer Cells and the Potential Significance of Metabolic Regulation in the Reversal of Cancer Chemoresistance

**DOI:** 10.3390/metabo10070289

**Published:** 2020-07-16

**Authors:** Xun Chen, Shangwu Chen, Dongsheng Yu

**Affiliations:** 1 Guangdong Provincial Key Laboratory of Stomatology, Department of Oral and Maxillofacial Surgery, Guanghua School of Stomatology, Sun Yat-sen University, Guangzhou 510055, China; chenx598@mail2.sysu.edu.cn; 2 Guangdong Key Laboratory of Pharmaceutical Functional Genes, MOE Key Laboratory of Gene Function and Regulation, State Key Laboratory for Biocontrol, Department of Biochemistry, School of Life Sciences, Sun Yat-sen University, Guangzhou 510275, China

**Keywords:** metabolic reprogramming, drug resistance, reversal of chemoresistance, metabolic regulation, chemoresistance

## Abstract

Metabolic reprogramming is one of the hallmarks of tumors. Alterations of cellular metabolism not only contribute to tumor development, but also mediate the resistance of tumor cells to antitumor drugs. The metabolic response of tumor cells to various chemotherapy drugs can be analyzed by metabolomics. Although cancer cells have experienced metabolic reprogramming, the metabolism of drug resistant cancer cells has been further modified. Metabolic adaptations of drug resistant cells to chemotherapeutics involve redox, lipid metabolism, bioenergetics, glycolysis, polyamine synthesis and so on. The proposed metabolic mechanisms of drug resistance include the increase of glucose and glutamine demand, active pathways of glutaminolysis and glycolysis, promotion of NADPH from the pentose phosphate pathway, adaptive mitochondrial reprogramming, activation of fatty acid oxidation, and up-regulation of ornithine decarboxylase for polyamine production. Several genes are associated with metabolic reprogramming and drug resistance. Intervening regulatory points described above or targeting key genes in several important metabolic pathways may restore cell sensitivity to chemotherapy. This paper reviews the metabolic changes of tumor cells during the development of chemoresistance and discusses the potential of reversing chemoresistance by metabolic regulation.

## 1. Introduction

**Figure 1:**
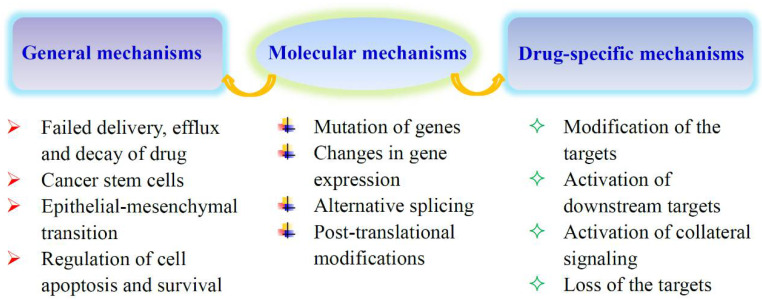
Mechanisms of acquired tumor drug resistance. Some of the reasons for acquired drug resistance are common to many drugs, and others may be drug-specific or pathway-specific. They are all related to the expression and function of specific genes. The pathway-specific mechanisms usually involve the restoration of the tumor-driving signaling pathway.

Chemotherapy and targeted therapy are common methods of tumor treatment, but tumor cells can develop resistance to drugs, leading to the failure of treatment. The development of tumor drug resistance involves general mechanisms and drug-specific or pathway-specific mechanisms (Figure 1) [1,2,3]. Pathway-specific mechanisms require the restoration of the tumor-driving signaling pathway. General mechanisms, common to many drugs, usually involve several aspects. First, the acquisition of resistance is often related to the failure of drug delivery. Multidrug resistance is mediated by the activation of ATP-binding cassette (ABC) transporters. The multidrug resistance protein 1 (MDR1) or P-glycoprotein is one of the members of ABC transporter family. The up-regulation of these proteins, especially MDR1, contributes to the acquired resistance to cytotoxic and targeted drugs. Drug escape can be rendered by cancer stem cells (CSCs). CSCs are a small population of tumor initiating cells that exist in tumors and are able to survive therapeutic intervention. The ability of CSCs resistance to chemotherapy attributes to their low proliferation rates, up-regulation of ABC transporter activity and DNA repair capacity, and down-regulation of apoptosis. Epithelial-mesenchymal transition (EMT) also contributes to the development of drug resistance. EMT is a transdifferentiation process in which epithelial cells lose their essential distinguishing characteristics and acquire instead mesenchymal features. Cells that undergo EMT can produce a CSC-like phenotype, leading to the resistance to tumor therapy. As most cancer drugs eradicate tumor cells by inducing apoptosis, the down-regulation of cell death pathways is another mechanism of drug resistance.

Metabolic reprogramming, metabolic alterations occurring in tumor cells, is one of the hallmarks of tumors [4]. Enhancement of cell proliferation in tumors requires metabolic reprogramming, which leads to the changes of metabolic pathways to generate enough ATP and intermediates for macromolecular biosynthesis under stress to meet the needs of rapid cell division. It is believed that metabolic reprogramming of tumor cells is the consequence of metabolic adaptation, which involves mutations in oncogenes, consequently dysregulating the expression and activity of some metabolic enzymes and the flux of metabolic pathways, and finally affecting whole cellular metabolism. Changes in cellular metabolism not only promote tumor development [5,6,7,8,9], but also contribute to cell resistance to chemotherapeutics [10,11,12,13]. These changes may result from the metabolic adaptation of tumor cells to pharmacological stress, which include adaptations in oxidative stress, lipid metabolism, and bioenergetics; dependence on glucose and glycolysis; and an increase in polyamine production, etc. [14,15,16,17]. Although the underlying mechanism of metabolic adaptation for drug resistant cells has not been well understood, emerging evidences suggest that expression of some metabolic genes or some signal pathways in the drug resistant cells are deregulated [18,19,20]. This leads to an increase in the uptake of glucose and glutamine by drug resistant cells [21,22]; activation of pathways such as glutaminolysis, glycolysis, and fatty acid oxidation [18,23,24]; and promotion of antioxidant production [22,25] as well as metabolic reprogramming in mitochondria [20].

The study on the effect of cell metabolism on tumor survival and growth revealed that tumor cells are more sensitive to nutrient deprivation and more dependent on some metabolites. Fasting or fasting-mimicking diets have been used as an auxiliary means to improve the outcome of cancer treatment [26]. A specific dietary manipulation will affect the metabolism of tumor cells, thus affecting the treatment of tumors [27]. In addition to in vivo evidence described above, in vitro experimental results show that targeting cell metabolism is a strategy to improve tumor therapy [28]. Preliminary studies in vitro have shown that regulating the level of metabolites or targeting some genes in certain metabolic pathways can make cancer cells re-sensitize to a chemotherapeutic agent [19,23,29,30,31]. This paper reviews the metabolic changes of tumor cells during the development of chemoresistance and discusses the possibility of reversing chemoresistance by metabolic regulation.

## 2. Metabolic Reprogramming Contributes to Chemoresistance

The metabolic response of drug resistant cancer cells is an open question. Metabolomics has been used to analyze the metabolic changes of drug resistant tumor cells in response to various chemotherapies. Available data imply that resistant cancer cells undergo metabolic adaptation in several aspects.

### 2.1. Oxidative Stress Adaptation

Antioxidant capacity and redox homeostasis are essential for cell survival and growth. Cisplatin is a kind of platinum compound commonly used in cancer treatment. The antitumor mechanism of platinum involves DNA damage-induced cell apoptosis and reactive oxygen species (ROS)-mediated oxidative stress [32,33,34]. Cisplatin resistant ovarian cancer cells have an active glutathione (GSH) synthesis pathway and high level of intracellular GSH (Table 1), suggesting that an increase of cellular GSH is a beneficial mechanism to cope with cisplatin induced oxidative stress [21,32]. For example, cisplatin resistant lung cancer cells increase ROS production through metabolic reprogramming and become more dependent on oxidative metabolism rather than glycolysis [35,36,37]. High levels of ROS and other metabolic alterations promote EMT [36]. These cells take up more glutamine and are highly sensitive to glutamine deprivation. Glutamine is converted into glutamate to synthesize GSH, consequently reducing the cellular ROS level. Blocking glutamate flux selectively kills cisplatin resistant cells [35]. Glutamate and glycine from GSH are mainly glutamine-derived, but cysteine is not [25]. The thiol component of GSH is cysteine. Cysteine uptake and lower level of cysteine endogenous synthesis are mandatory for GSH bioavailability. The thiols from GSH as well as cysteine–glycine and cysteine can confiscate platinum molecules, avoiding them to reach cell components and form protein and DNA adducts [25]. The cysteine is essential for cancer proliferation and survival, and critical for metabolic rewiring of cancer [38,39,40,41,42]. Cysteine impacts the hypoxic adaptation of cancer cells, thus contributing to hypoxia-driven platinum-based chemotherapeutic agents’ resistance. Therefore, regulating cysteine bioavailability may serve as strategy to reverse resistance both to hypoxia and carboplatin [39,40]. Cysteine is also a valuable source of pyruvate for cancer cells replacing in part glucose; cysteine catabolism generates about 20% of intracellular pyruvate [41].

In addition to cisplatin, other antitumor agents also induce the changes of cellular GSH level. Paclitaxel (Taxol) targets cellular tubulin, which leads to defects in mitotic spindle assembly, chromosome segregation, and cell division. In triple negative breast cancer cells treated with paclitaxel, the levels of glutamine, glutamate, and GSH increase [43]. Sorafenib is a tyrosine kinase inhibitor. GSH synthesis in sorafenib resistant leukemia cells was enhanced, indicating that the resistant cells had specific metabolic and redox adaptations [17]. Erlotinib, an epidermal growth factor receptor (EGFR) inhibitor, is used for the therapy of mutated EGFR-driven cancers. The secondary mutation of T790M EGFR induces the acquisition of drug resistance in tumors. However, the metabolomic profiles of erlotinib sensitive and resistant cells showed that GSH levels are significantly down-regulated in T790M EGFR cells. The T790M mutation inhibits NFE2-related factor 2 (NRF2) activity, which down-regulates the expression of GSH-synthesizing enzymes [44]. Supplement of GSH in resistant cells re-sensitized them to erlotinib. In contrast, reducing GSH levels in sensitive cells made them resistant to erlotinib. Increasing intra-tumoral GSH levels re-sensitized resistant tumors to erlotinib in mice. Because the drug resistance of tumors is usually related to the increase of GSH level, the reason the decrease of GSH level is related to the resistance of EGFR tyrosine kinase inhibitor deserves further study.

Glucose was channeled to the pentose phosphate pathway (PPP) and serine synthesis pathway in bortezomib resistant cells, which increased antioxidant capacity of bortezomib resistant cells [49]. Bortezomib is a proteasome inhibitor. Proteasome inhibition causes endoplasmic reticulum stress and activation of the unfolded protein response, eventually leading to apoptosis through multiple pathways, including overproduction of ROS. The PPP can produce reduced form of nicotinamide adenine dinucleotide phosphate (NADPH), consequently maintaining intracellular GSH level and redox balance. High activity of serine synthesis pathway is attributed to the up-regulation of 3-phosphoglycerate dehydrogenase (PHGDH), the rate-limiting enzyme of serine synthesis. PHGDH decreased ROS through increasing GSH synthesis, thereby promoting cell growth and bortezomib resistance [50].

### 2.2. Adaptation of Lipid Metabolism

Increasing evidence suggests that lipid metabolism is reprogrammed when tumor cells respond to chemotherapy [51]. Early studies have shown that compared with cisplatin sensitive ovarian cancer cells, cisplatin resistant counterparts have higher basal content of intracellular mobile lipids originating from the hydrolyzed acyl chains in triacylglycerides (Table 1) [21,52,53]. Similar reprogramming in lipid metabolism was conformed in cisplatin resistant bladder cancer through a comparative lipidomic profiling [54]. The metabolomic analysis of lung cancer cells and their cisplatin resistant derivative showed that there were 40 differential metabolites, mainly involving phospholipids, fatty acids, amino acids, and metabolites related to energy metabolism [55]. The analysis of five metabolite groups showed that the level of short-chain acylcarnitines and selected lysophosphatidylcholines increased and the level of acyl-alkyl-phosphatidylcholines and one sphingolipid decreased in the erlotinib resistant human pancreatic cancer cells compared with the erlotinib sensitive parental cells. The results suggested that choline phospholipid and acetyl-CoA-associated metabolism was changed after the acquisition of erlotinib resistance in pancreatic cancer [16]. The content of choline was higher in docetaxel resistant BRCA1-mutated mouse mammary tumor tissues compared with the docetaxel sensitive controls [47]. Temozolomide is a chemotherapeutic drug that serves as a DNA alkylator. Temozolomide treatment showed up-regulation of choline and phosphorylcholine in temozolomide sensitive glioblastoma multiforme (GBM) cells compared to temozolomide resistant GBM cells (Table 1) [48]. With some exceptions, the increase of phospholipid appears to be related to cell resistance.

### 2.3. Bioenergetic Adaptation

Altered energetics is considered a hallmark of cancer. Bioenergetic adaptation is also a mechanism for cancer cells to cope with drug challenges. The cisplatin sensitive ovarian cancer cells have active glycolysis. However, their cisplatin resistant counterparts can enhance the glycolytic flux upon the treatment with ATP synthase inhibitor or gain greater oxidative phosphorylation (OXPHOS) compensation in the presence of glycolysis inhibition. The results indicated that resistant ovarian cancer cells are able to switch between OXPHOS and glycolysis [56]. Resistant chronic myelogenous leukemia (CML) cells elevated creatine production and creatine conversion to phosphocreatine [46]. The increase of phosphocreatine levels may provide an alternative energy reserve, allowing cells to escape imatinib-induced cell death [57].

### 2.4. Dependence on Glucose and Glycolysis

Tumor cells prefer to fuel glucose to the aerobic glycolysis pathway. Sorafenib is a tyrosine kinase inhibitor. Metabolism in sorafenib resistant leukemia cells was reprogrammed to increase glucose demand and decrease the flux of glucose into the PPP [17]. Imatinib is a breakpoint cluster region gene-Abelson murine leukemia viral oncogene homolog (BCR-ABL) tyrosine kinase inhibitor, and the metabolic changes of CML cells resistant to imatinib are contradictory. An early study found that compared with imatinib sensitive cells, the imatinib resistant BCR-ABL-positive cells increased glucose uptake, glycolysis, and lactate production in response to the treatment of imatinib [45]. However, Dewar et al. revealed that resistant CML cells reduced glucose consumption and lactate production [46]. The reasons for the inconsistent results are not clear. Flavopiridol is a pan-cyclin dependent kinase inhibitor inducing cancer cell apoptosis. Flavopiridol resistant human prostate cancer cells showed enhanced glycolysis and were less sensitive to the apoptosis induced by cisplatin and docetaxel [58]. Adriamycin (doxorubicin) is a topoisomerase II inhibitor. Compared with the sensitive cells, the central metabolism of adriamycin resistant leukemia cells has changed, which is characterized by increased dependence on glucose, decreased dependence on exogenous glutamine, and decreased pantothenic acid uptake and fatty acid β oxidation rate [59].

### 2.5. Synthesis of Polyamine

Polyamines are ubiquitous in living cells and are essential for eukaryotic cell growth [60]. Polyamine synthesis was reported as one of most significant metabolic changes in platinum resistant ovarian cancer cells [32]. Metabolomics analysis of the supernatant of tumor associated macrophages (TAMs) in colorectal cancer demonstrated that 5-Fluorouracil (5-FU) could stimulate the secretion of putrescine that induced the resistance of tumor cells to 5-FU-mediated apoptosis. Inhibition or down-regulation of ornithine decarboxylase, a key enzyme in the production of putrescine, reduced putrescine levels, thus preventing the TAMs-mediated 5-FU resistance and enhancing the 5-FU-induced growth inhibition of cancer cells [15].

Current evidence supports the existence of metabolic reprogramming in drug resistant cancer cells. It has been found that drug sensitive and resistant cancer cells respond differently to certain drugs [48,61]. The metabolic pattern of adriamycin sensitive cells is gradually similar to that of adriamycin resistant cells, which further supports a metabolic shift in the development of chemoresistance [61]. The metabolic changes induced by chemotherapy may be a dynamic response [62,63]. A short-term treatment of gastric cancer cells with 5-FU can reduce cellular proline and increase glutamate, which may be associated with the up-regulation of proline dehydrogenase (PRODH) that promotes production of glutamate from proline [63]. But the proline and glutamate levels are less affected in 5-FU resistant cells, where PRODH expression was not up-regulated following 5-FU treatment. PRODH catalyzes the first step of proline degradation, inducing superoxide and conversion of proline to glutamate. The inhibition of nucleotide synthesis by 5-FU and the resultant genetic stress induces PRODH activity following mitochondrial superoxide generation, contributing to drug resistance. In general, metabolic adaptation of resistant cancer cells may involve many aspects, including redox, lipid metabolism, bioenergetics, glycolysis, and synthesis of polyamine. The diversified antitumor mechanisms of different chemotherapeutic agents may lead to different resistance-related metabolic alterations. However, some drugs may induce similar metabolic change. The cells resistant to adriamycin and the cells resistant to cisplatin shared some similar differential metabolites compared with their drug sensitive counterparts [2]. In addition, the treatment of cells with different drug combinations may lead to different metabolic alterations. For example, the treatment of human breast cancer cells with a combination of cisplatin and tamoxifen significantly reduces cellular phosphocholine levels, while cisplatin combined with adriamycin can increase lactate levels [11]. Although prediction of such metabolic changes is still difficult, some studies have tried to identify the plasma markers from cancer patients for predicting drug resistance [64].

## 3. The Potential Mechanisms of Metabolic Alterations in Resistant Cells

**Figure 2:**
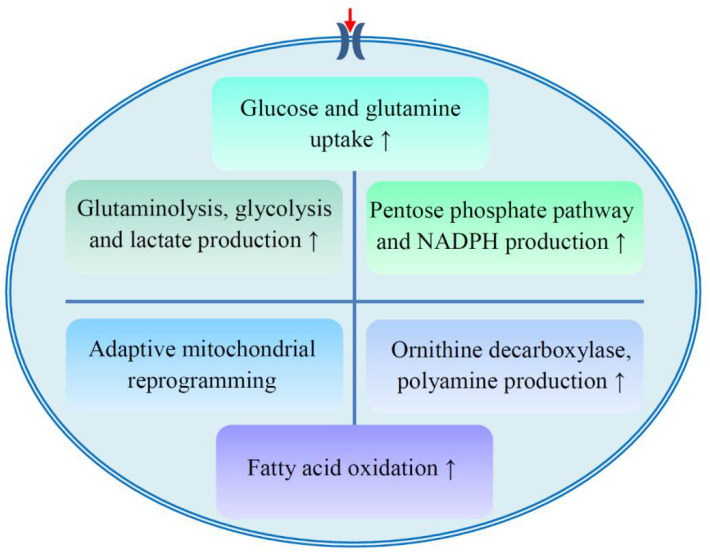
Proposed metabolic mechanisms of drug resistance. The development of drug resistance involves several aspects of metabolism, including the enhancement of glucose and glutamine uptake; active glutaminolysis, glycolysis, pentose phosphate pathway, and fatty acid oxidation; polyamine production; and adaptive mitochondrial reprogramming. NADPH, reduced form of nicotinamide adenine dinucleotide phosphate.

We know that several metabolic enzymes, such as glycolytic enzymes, are targets for tumor therapy [65,66]. Many studies have explored the underlying mechanism of metabolic reprogramming in cancer cells, but the mechanism of metabolic adaptation in drug resistant cells is still unclear. Several potential mechanisms are proposed to explain metabolic reprogramming and drug resistance (Figure 2, Table 2).

First, the uptake of glucose and glutamine in resistant cells increases [21,22,24,67,68]. The ibrutinib resistant cells relied heavily on exogenous glutamine [24], and cisplatin resistant ovarian cancer cells increased glucose uptake and consumption [21,22]. Mucin1, a glycoprotein, was associated with chemoresistance and cancer aggression. Over-expression of mucin1 promoted glucose and glutamine uptake in breast cancer [68]. The expression of glucose transporter protein 1 (GLUT1) is up-regulated in oral cancer cells under hypoxia. Silencing of GLUT1 resulted in increased rates of cisplatin-induced apoptosis under hypoxia [67].

However, fatty acid oxidation is important for the resistance of certain cells (Figure 2, Table 2). Upon treatment of ibrutinib plus etomoxir, the carnitine palmitoyltransferase I (CPT-1) inhibitor suppressing β-oxidation of fatty acids in mitochondria, GLUT1 expression and glucose uptake in resistant chronic lymphocytic leukemia (CLL) cells were decreased compared to sensitive cells, suggesting the activation of fatty acid oxidation might be important for the sustenance of ibrutinib resistance in CLL cells [24].

Resistant cells exhibit active glutaminolysis and glycolysis pathways and increase lactate production (Figure 2, Table 2) [18,23,30,69,70]. The catabolism of glutamine in ibrutinib resistant cells was up-regulated [24]. Glutaminolysis was associated with the resistance of ovarian cancer cells to paclitaxel or cisplatin [23]. The expression of lactate dehydrogenase A (LDHA) in paclitaxel resistant cells increased [30]. Glycolytic enzymes, enolase and glyceraldehyde-3-phosphate dehydrogenase (GAPDH), are the resistance-related proteins of carboplatin and paclitaxel [18]. Enolase was also up-regulated in cisplatin resistant gastric cancer cells [70]. Melanoma cell adhesion molecule (MCAM), a cell surface receptor, was identified as being significantly up-regulated in chemoresistant lung cancer cells and patient-derived xenografts. Metabolomic profiling revealed that MCAM regulated lactate production and chemosensitivity of resistant cells through PI3K/AKT/SOX2 pathways [69].

Resistant cells channeled glucose to PPP and promoted the production of NADPH (Figure 2, Table 2) [19,22,24]. Glucose-6-phosphate dehydrogenase (G6PDH) and 6-phosphogluconate dehydrogenase (6PGD) are two important enzymes in PPP. The expression of G6PDH in cisplatin resistant cells was up-regulated [22]. 6PGD activity was related to sensitivity of hepatocellular carcinoma (HCC) to paclitaxel, adriamycin, and cisplatin [19]. PPP-mediated production of NADPH is beneficial for tumor cells to cope with chemotherapy-induced oxidative stress. Hepatocyte nuclear factor 1β (HNF1β) regulates γ-glutamylcysteine ligase expression and GSH production in ovarian cancer cells. HNF1β down-regulation sensitizes cells to carboplatin [25].

Resistant cells may have adaptive mitochondrial reprogramming (Figure 2, Table 2) [20,71,72,73]. PI3K therapy (targeting PI3K) induces global metabolic reprogramming in tumors and promotes the recruitment of Akt2 to mitochondria, which facilitates mitochondrial energy metabolism and reduces tumor cell death, conferring resistance to PI3K therapy [20]. Adenylate kinase 4 (AK4) is one of the key enzymes that catalyze the high-energy phosphoryl transfer reaction in mitochondria. AK4 knockdown increased the levels of fumarate and malate in tricarboxylic acid (TCA) cycle intermediates and enhanced the sensitivity of tumor cells to cisplatin and hypoxia. This may be due to its regulation of mitochondrial activity, including the increase of mitochondrial number and the up-regulation of gene expression of key enzymes in the TCA cycle, succinate dehydrogenase A, and oxoglutarate dehydrogenase L [71]. Tumor necrosis factor-associated protein 1 (TRAP1) facilitated a metabolic shift toward OXPHOS in ovarian cancer, which is related to cisplatin resistance [72].

Production of polyamines also contributes to chemoresistance (Figure 2, Table 2). Metabolic pathways of polyamines as well as amino acids and fatty acids were significantly changed in erlotinib resistant pancreatic cancer cells. Putrescine, the product catalyzed by ornithine decarboxylase (ODC), contributed to the acquisition of erlotinib resistance. ODC inhibition was able to restore erlotinib sensitivity in erlotinib resistant cells, which could be rescued by exogenous putrescine [31].

The mechanisms proposed may partly explain the metabolic adaptation during the development of drug resistance. One or several mechanisms may be used by cells to cope with a drug. Little is known about the correlation between the antitumor mechanism of a drug and its principle of metabolic adaptation, which is worth further study.

## 4. Potential Reversal Effect of Metabolic Regulation on Chemoresistant Tumor Cells

**Figure 3:**
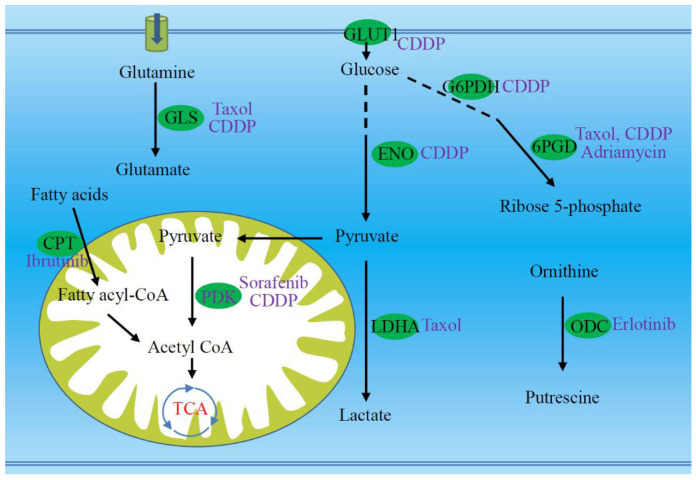
Targeting several key enzymes or proteins (green ellipses) are potential to re-sensitize the resistant tumor cells to chemotherapeutic agents (purple font). Targets marked with green ellipses involve multiple metabolic pathways, including glutaminolysis, transport and glycolysis of glucose, pentose phosphate pathway, fatty acid oxidation, pyruvate dehydrogenation, and production of polyamine. The drugs that can be metabolically regulated are highlighted with purple font. 6PGD, 6-phosphogluconate dehydrogenase; CDDP, cisplatin; CoA, coenzyme A; CPT, carnitine palmitoyltransferase; ENO, enolase; G6PDH, glucose-6-phosphate dehydrogenase; GLS, glutaminase; GLUT1, glucose transporter protein 1; LDHA, lactate dehydrogenase A; ODC, ornithine decarboxylase; PDK, pyruvate dehydrogenase kinase.

Since the development of chemoresistance is associated with metabolic reprogramming, it should be possible to restore the chemosensitivity through metabolic regulation (Figure 3). Some researchers have tried to do this and have achieved initial success. Metabolic regulation on resistant tumor cells may restore the cell sensitivity to chemotherapy [30].

First, metabolic regulation on glutamine in resistant cancer cells re-sensitizes their sensitivity to chemotherapeutics. It has been well known that cancer cells increase glutamine uptake, and even develop a reliance on glutamine. Cancer cells usually up-regulate the expression of glutaminase that catalyzes the first step of glutamine degradation (glutaminolysis). The treatment of paclitaxel or cisplatin resistant ovarian cancer cells with a glutaminase inhibitor increased their sensitivity to chemotherapy through inhibiting cell proliferation, regardless of their glutamine dependence status [23]. The expression of glutaminase 1 (GLS1) was enhanced in metastatic glutamine-dependent ovarian cancer cells and targeting GLS1 using siRNA sensitized the cancer cells to cisplatin (Figure 3). Argininosuccinate synthetase 1 (ASS1) is a chemosensitivity marker for arginine and glutamine starvation therapy. Up-regulation of ASS1 expression increased the resistance to glutamine starvation therapy. The supplement of fumarate suppressed ASS1 expression, which sensitized tumor cells to arginine and glutamine deprivation therapy [29].

Down-regulation of the glycolysis pathway in resistant cells may restore the cell sensitivity to chemotherapy. Cisplatin resistant gastric cancer cells highly depend on glycolysis through metabolic reprogramming. Disturbing cellular glycolysis via glucose starvation or 2-deoxyglucose treatment significantly reversed drug resistance. Enolase 1 is a glycolytic enzyme, and its up-regulation promotes the resistance of gastric cancer cells to cisplatin. Knockdown of enolase 1 reduced glycolysis and reversed drug resistance [70]. In paclitaxel resistant breast cancer cells, the expression and activity of LDHA increased compared with its parental cells. Silencing LDHA expression or inhibiting its activity re-sensitized the resistant cells to paclitaxel [30]. Pyruvate dehydrogenase kinase 4 (PDK4) is an inhibitor of mitochondrial pyruvate dehydrogenase, and inhibiting PDK4 reversed the resistance of HCC stem cells to sorafenib or cisplatin (Figure 3) [73].

Down-regulation of antioxidant capacity of resistant cells also contributes to the re-sensitivity of cells to drugs. Inhibition of GSH production by buthionine sulphoxamine (BSO) sensitizes ovarian clear cell carcinoma cells to carboplatin [25]. The dehydrogenation of glucose-6-phosphate and 6-phosphogluconate is two critical steps in the oxidative phase of PPP, in which NADPH is produced. The cisplatin resistant cells up-regulate expression and enzymatic activity of G6PDH and are more sensitive to G6PDH inhibition, suggesting that PPP is a potential target to overcome cisplatin resistance. Targeting G6PDH sensitized cisplatin resistant cells (Figure 3) [22]. 6PGD was found to be important for HCC growth and survival. 6PGD inhibition sensitized HCC to chemotherapy of paclitaxel, adriamycin, and cisplatin via AMP-activated protein kinase (AMPK)-dependent NADPH metabolic suppression and oxidative stress, suggesting that 6PGD is a promising therapeutic target to overcome chemoresistance [19]. In addition, serine starvation increased the sensitivity of multiple myeloma cells to bortezomib, suggesting the importance of serine metabolism in the cell response to bortezomib [49].

Fatty acid oxidation is another regulatory point. Carnitine palmitoyltransferase catalyzes the transport of long chain fatty acids into mitochondria for oxidation. Inhibition of carnitine palmitoyltransferase re-sensitized resistant leukemia cells to the tyrosine kinase inhibitor ibrutinib, suggesting that slowing down fatty acid oxidation may overcome ibrutinib resistance [24].

Of course, the regulation of drug degradation and excretion will affect the drug resistance of cells. Glucuronidation of drugs by uridine diphosphate-glucuronosyltransferases (UGT1As) contributes to the resistance of cancer cells to antitumor drugs. Selective UGT1As inhibitors specifically restored sensitivity of resistant cancer cells [74].

Several natural compounds have been shown to reverse chemoresistance [75,76,77]. Ursolic acid (UA) is a natural compound with antitumor activity. When combined with adriamycin, it can reverse the multidrug resistance of breast cancer cells, which is attributed to the changes of energy metabolism and amino acid metabolism induced by UA [76]. Treatment with UA reduces the level of intracellular metabolites, including lactate, pyruvate, glucose, α-ketoglutarate, and several amino acids. β-elemene, extracted from *Curcuma zedoaria Roscoe*, is potential to reverse the resistance to erlotinib [78]. Melittin, a main peptide with a potential for anticancer activity in bee venom, has a synergistic effect with cisplatin on ovarian cancer cells in several key metabolic pathways, such as TCA cycle, OXPHOS, purine and pyrimidine metabolism, and the arginine/proline pathway [77].

## 5. Conclusions

Drug resistance is the determinant of chemotherapy failure. The metabolic adaptability of cancer cells has been linked to their resistance to antitumor drugs. Resistant cells adapt to oxidative stress and maintain intracellular redox homeostasis by increasing the production of GSH and NADPH. Resistant cells can also increase fatty acid oxidation and phospholipid biosynthesis to obtain sufficient energy supply and maintain the integrity of cell membrane. Bioenergetic adaptation provides an alternative energy for resistant cells to survive drug intervention. Some resistant cells are highly dependent on glucose and glutamine. Glycolysis and glutaminolysis provide energy and intermediates for cell proliferation. Polyamines have essential roles in cell proliferation [79]. TAMs in tumors can secrete putrescine to induce drug resistance of tumor cells. According to different chemotherapeutic agents, resistant cells may adopt different adaptive mechanisms. Metabolic adaptation can be attributed to the mutation or deregulation of some genes, which lead to increased glucose and glutamine uptake, enhanced glutaminolysis and glycolysis pathways, boosted pentose phosphate pathway and antioxidant capacity, adaptive mitochondrial reprogramming, etc. This also provides an option to re-sensitize resistant cells to chemotherapy by regulating cell metabolism. There are some successful examples for the reversal of chemoresistance, which aim to target some key genes in the metabolic pathway or regulate the supply of metabolites.

There are two issues to be addressed here. First, many data discussed above are obtained by two-dimensional cell culture of established cell lines, with little regard for the untransformed state and the role of cell–cell interactions that modulate gene expression and enzyme activity. This has led to some observations that are true in cell culture, but do not always hold up in more complex models. Therefore, it is necessary to adopt different research models, such as ganoids and animal models, and combine with clinical data. Since metabolism does not operate in isolation from gene expression, mutational events, and the subsequent protein activities, more use of other ‘omics’ technologies such as transcriptomics and proteomics will help to understand the potential mechanism of metabolic reprogramming in response to chemotherapy. It also helps to assess whether the altered metabolism observed is a driver or a bystander. Another concern is the diverse metabolic responses to chemotherapeutics, which are affected by many factors. Glutathione levels increased in response to low dose curcumin in breast cancer cells, but decreased at high dose, indicating a biphasic response [80]. Triple-negative and hormone receptor-positive breast cancer cells had different metabolic responses to paclitaxel [43]. Comparable tumors, such as those from the same source, may respond differently to certain chemotherapy [11]. Better understanding of drug resistance-related metabolic reprogramming may help to develop new therapeutic strategies [57,59].

## Figures and Tables

**Table 1:** Main metabolic changes of chemoresistant tumor cells.

Samples	Analysis Approach	Drugs	Metabolic Changes	Reference
Ovarian cancer cells	NMR	Cisplatin	GSH, mobile lipids↑	[21]
Lung cancer cells	Fluorimeter	Cisplatin	ROS↑	[35]
ESCC cells	NMR	5-Fluorouracil	Glutamate↓, lactate, glutamine↑	[14]
TAMs	GC-TOF-MS	5-Fluorouracil	Putrescine↑	[15]
Pancreatic cancer cells	MS	Erlotinib	Short-chain acylcarnitines, lysophosphatidylcholines↑, acyl-alkyl-phosphatidylcholines, sphingolipid↓	[16]
Lung cancer cells	NMR	Erlotinib	GSH↓	[44]
Leukemia cells	UHPLC-ESI-MS	Sorafenib	GSH↑	[17]
CML cells	NMR, GC-MS	Imatinib	Lactate ↑	[45]
CML cells	NMR	Imatinib	Creatine, phosphocreatine↑, lactate↓	[46]
Breast cancer cells	NMR	Paclitaxel	Glutamine, glutamate, GSH↑, lysine, proline, valine↓	[43]
Mouse mammary tumor tissues	NMR	Paclitaxel	Choline↑	[47]
Glioblastoma multiforme cells	NMR	Temozolomide	Alanine, choline, creatine, and phosphorylcholine↓	[48]

CML, chronic myelogenous leukemia; ESCC, esophageal squamous cell carcinomas; GC-TOF-MS, gas chromatography and time-of-flight mass spectrometer; GSH, glutathione; MS, mass spectrometer; NMR, nuclear magnetic resonance; ROS, reactive oxygen species; TAMs, tumor associated macrophages; UHPLC-ESI–MS, ultra-high-performance liquid chromatography electrospray ionization mass spectrometry. ↑, increase; ↓, decrease.

**Table 2:** Potential metabolic mechanisms of drug resistance.

Events.	Examples	References
Glucose and glutamine uptake	Mucin1 promotes glucose and glutamine uptake, which is associated with chemoresistance; the ibrutinib resistant cells absorb more glutamine; down-regulation of GLUT1 reduces the sensitivity of oral cancer cells to cisplatin.	[21,22,24,67,68]
Glutaminolysis, glycolysis and lactate production	MCAM regulates lactate production and chemosensitivity; enolase and GAPDH are carboplatin and paclitaxel resistance-related proteins; knockdown of enolase reverses cisplatin resistance; LDHA regulates the sensitivity of resistant cells to paclitaxel; glutaminase inhibitor increases the sensitivity of resistant cells to paclitaxel or cisplatin.	[18,23,30,69,70]
PPP and NADPH production	Expression and enzymatic activity of G6PDH are up-regulated in cisplatin resistant cells; 6PGD inhibition increases the sensitivity of HCC to paclitaxel, adriamycin, and cisplatin; NADPH accumulates in ibrutinib resistant cells.	[19,22,24]
Adaptive mitochondrial reprogramming	Resistance to PI3K therapy is related to adaptive mitochondrial reprogramming; AK4 knockdown increases the sensitivity to cisplatin; TRAP1 facilitates a metabolic shift toward OXPHOS, which is related to cisplatin resistance; PDK4 inhibition reverses the resistance of HCC stem cells to sorafenib or cisplatin.	[20,71,72,73]
Fatty acid oxidation	Inhibition of fatty acid oxidation re-sensitizes resistant cells to ibrutinib.	[24]
ODC and polyamine production	ODC-mediated production of putrescine contributes to the acquisition of erlotinib resistance.	[31]

6PGD, 6-phosphogluconate dehydrogenase; AK4, Adenylate kinase 4, G6PDH, glucose-6-phosphate dehydrogenase; GAPDH, glyceraldehyde-3-phosphate dehydrogenase; GLUT1, glucose transporter protein 1; HCC, hepatocellular carcinoma; LDHA, lactate dehydrogenase A; MCAM, melanoma cell adhesion molecule; ODC, ornithine decarboxylase; PDK4, pyruvate dehydrogenase kinase 4; PPP, pentose phosphate pathway; TRAP1, tumor necrosis factor-associated protein 1.
